# Intravenous Anesthetic Protects Hepatocyte from Reactive Oxygen Species-Induced Cellular Apoptosis during Liver Transplantation *In Vivo*

**DOI:** 10.1155/2018/4780615

**Published:** 2018-10-28

**Authors:** Weifeng Yao, Xue Han, Yihan Zhang, Jianqiang Guan, Mian Ge, Chaojin Chen, Shan Wu, Jiaxin Chen, Gangjian Luo, Pinjie Huang, Ziqing Hei

**Affiliations:** ^1^Department of Anesthesiology, Third Affiliated Hospital, Sun Yat-sen University, Guangzhou, Guangdong 510630, China; ^2^Department of Anesthesiology, Sun Yat-sen Memorial Hospital, Sun Yat-sen University, Guangzhou 510000, China

## Abstract

**Background:**

Liver transplantation leads to liver ischemia/reperfusion (I/R) injury, resulting in early graft dysfunction and failure. Exacerbations of oxidative stress and inflammatory response are key processes in the development of liver I/R injury. Intravenous anesthetic propofol potent effects on free radical scavenging and protects livers against I/R injury. However, the role and mechanism of propofol-mediated hepatic protection in liver transplantation is poorly understood. The aim of this study was to evaluate the role of propofol postconditioning in the liver I/R injury after liver transplantation.

**Methods:**

Forty-eight rats were randomly divided into six groups: rats receiving either sham operation or orthotopic autologous liver transplantation (OALT) in the absence or presence of propofol (high dose and low dose) postconditioning or intralipid control or VAS2870 (Nox2 special inhibitor). Eight hours after OALT or sham operation, parameters of organ injury, oxidative stress, inflammation, and NADPH-associated proteins were assessed.

**Results:**

After OALT, severe liver pathological injury was observed that was associated with increases of serum AST and ALT, which were attenuated by propofol postconditioning. In addition, especially high dose of propofol postconditioning reduced TNF-*α*, IL-1*β*, IL-6, TLR4, and NF-*κ*B inflammatory pathway, accompanied with decrease of neutrophil elastase activity, MPO activity, 8-isoprotane, p47^phox^ and gp91^phox^ protein expressions, and increase of SOD activity. Inhibition of Nox2 by VAS2870 conferred similar protective effects in liver transplantation.

**Conclusion:**

Liver transplantation leads to severe inflammation and oxidative stress with NADPH oxidase activation. Propofol postconditioning reduces liver I/R injury after liver transplantation partly via inhibiting NADPH oxidase Nox2 and the subsequent inflammation and oxidative stress.

## 1. Introduction

Liver transplantation has become the effective surgical treatment for patients with end-stage liver disease [1]. Liver ischemia reperfusion (I/R) injury is a severe postoperative complication during the early period after transplantation. It leads to early graft dysfunction and failure, which further results in acute and chronic rejection and irreversible death [2]. Characterized by uncontrolled inflammatory response, liver I/R injury promotes hypoxic hepatocyte reoxygenation and reactive oxygen species (ROS) formation, which result in neutrophil infiltration, robust ROS generation, and ultimately programmed death of hepatocytes [3]. To date, the mechanisms accounted for liver injury, especially I/R injury during liver transplantation, are complicated and remained unclear; strategies for preventing I/R injury are still lacking. Hence, seeking protective intervention of I/R injury during perioperative period is regarded urgent and profound scientific significance and important clinical applications.

Propofol (2,6-diisopropylphenol) is widely used for anesthesia induction and maintenance during perioperation and sedation in intensive care unit (ICU) patients [4]. In addition to its clinical usages, propofol exerts anti-inflammatory and antioxidative effectiveness basing on the chemical group phenol [5]. In our previous study, we found that propofol pretreatment could attenuate pulmonary oxidative stress induced by liver transplantation through activating Nrf2 nuclear translocation and upregulating its downstream of HO-1 antioxidant enzyme formation [6, 7]. However, in most clinical situations, propofol pretreatment is not feasible while treatment at the onset or after induction of reperfusion, termed as postconditioning, is more applicable [8], which has been proven as a promising therapeutic strategy against ischemia/reperfusion damage. Whether propofol postconditioning is crucial in reducing oxidative stress in liver I/R injury and the underline mechanism remains unknown.

Nicotinamide adenine dinucleotide phosphate (NADPH) oxidase (Noxs) is one of the major sources of cellular ROS, which has been identified to play an important role in liver I/R injury [9]. Nox2 and Nox4 are two predominant Nox isoforms existing in hepatocytes in liver parenchyma [10]. Nox2-deficient mice showed lower mortality rate than wild type group when subjected to hepatic I/R injury. Whether Nox2 is correlated to liver transplantation-induced hepatic oxidative stress and contributed to the antioxidant property of propofol need to be verified.

Therefore, the current study observed the effects of propofol postconditioning on liver I/R injury induced by liver transplantation and further explored the potential mechanism whether the protective effects provided by propofol postconditioning are associated with Nox2-related oxidative stress pathway.

## 2. Materials and Methods

### 2.1. Experimental Protocols

Male Sprague-Dawley rats (220–250 g, 8 weeks) obtained from Medical Experimental Animal Center of Guangdong Province (Guangzhou, China) were housed in the animal room of Zhongshan Medical School (Guangzhou, China). Rats were fasted for 8 hours prior to the study but were allowed to access tap water ad libitum. All the animal care and research protocols were approved by the Institutional Animal Care and Use Committee of Sun Yat-sen University (Guangzhou, China) and performed in accordance with National Institutes of Health guidelines for the use of experimental animals.

The orthotopic autologous liver transplantation (OALT) model was carried out according to our previous study [11, 12]. Rats were randomly divided into six groups (*n* = 8) as follows: sham-operated control (sham) and OALT, OALT treated with intralipid (OALT + INT), OALT treated with high dose of propofol (OALT + HPro), OALT treated with low dose of propofol (OALT + LPro), and OALT treated with VAS2870 (OALT + VAS). High dose (40 mg/kg/h) or low dose (20 mg/kg/h) of propofol [13] or the same volume of intralipid was administrated continuous via tail vein for 30 min at the onset of reperfusion. Some of the rats were treated with specific Nox2 inhibitor VAS2870 (2 mg/kg, Sigma, USA) [14] intravenously after reperfusion.

### 2.2. Sample Harvest

Blood and liver samples were harvested eight hours after reperfusion. Under general anesthesia, animals were euthanized by a lethal injection of sodium pentobarbital. The blood was collected from carotid artery into heparinized tubes and then centrifuged for 15 min at 2000*g* (4°C). The supernatants were collected and stored at −80°C until measurement. Median hepatic lobes were immediately and promptly taken out (about 0.5 cm^3^), washed in cold saline, fixed in 10% formalin solution, dehydrated in ascending grades of alcohol, and then embedded in paraffin. The residual parts of liver tissue were harvested and stored at −80°C until further measurement.

### 2.3. Serum Aspartate Aminotransferase (AST) and Alanine Aminotransferase (ALT) Levels

The activity of AST and ALT in serum, indicators of liver cellular damage, was measured by a clinical chemistry analyzer system.

### 2.4. Histological Examination of Liver Sections

Median hepatic lobes were fixed in 4% buffered formalin. After embedding and cutting of 4 *μ*m slices, all samples were stained with hematoxylin/eosin. The staining sections were visualized and images were acquired using a microscope with 10x and 40x objectives. Histological evaluation was performed in a blinded manner. The severity of liver injury was graded with modified Suzuki criteria [15].

### 2.5. Assay of Inflammatory Cytokines and Oxidative Stress Markers

Part of the liver was homogenized with a Potter liver homogenizer at 500 g and centrifuged at 800*g* for 10 min. The supernatant was pipetted into a fresh Eppendorf cup for the detection of cytokines. Inflammatory cytokines including TNF-*α*, IL-1*β*, and IL-6 in the liver were quantified with commercial ELISA kits (KeyGen BioTech, China). And the neutrophil elastase and 8-isoprostane were detected using ELISA kits (Cayman Chemical). Superoxide dismutase (SOD) and myeloperoxidase (MPO) activities were measured according to our previous methods [16].

### 2.6. Immunohistochemical Staining of p47^phox^

Immunohistochemical staining was performed in a previous study [16]. Liver tissue sections were deparaffinized, hydrated, and incubated at 4°C overnight with a p47^phox^ primary antibody (Cell Signaling Technology, Danvers, USA, diluted 1 : 500), followed by incubation with a horseradish peroxidase-coupled anti-rabbit IgG secondary antibody at room temperature for 2 hours and then colored with diaminobenzidine (DAB) for 3 minutes. Phosphate-buffered saline (PBS) was used to replace primary antibody in the negative control. DAB staining intensity was observed under light microscope (Leica Microsystems Digital Imaging, Cambridge, UK) and assessed with a microscopic image analysis system (ImageJ, National Institutes of Health, USA).

### 2.7. TUNEL for DNA Fragmentation

The nuclear DNA fragmentation, a specific biochemical hallmark of apoptosis, was labeled by TUNEL staining with a Dead End Fluorometric TUNEL system kit (Promega Corp., Madison, Wisconsin, USA) according to the manufacturer's protocol and our previous study [17]. Liver sections were incubated with proteinase K solution (20 *μ*g/ml in PBS) at room temperature for 10 minutes after deparaffinization and hydration. TUNEL labeling was conducted with a 100 *μ*l mix of equilibration buffer, nucleotide mix, and recombinant terminal deoxynucleotidyl transferase (rTdT) enzyme (the volume ratio was 45 : 5 : 1) in a humidified, lucifugal chamber for 1 hour at 37°C, from which step to the end of experiment, the slides were protected from direct light. The reaction was terminated by immersing the slides in a 2 × SSC buffer for 15 minutes at room temperature and then were rinsed with PBS. Nuclear was visualized by DAPI staining. The liver tissues were covered by an antifade solution and mounted by glass coverslips with clear nail polish sealing the edges. The slides were immediately analyzed by a fluorescence microscope and then stored at −20°C in dark if necessary.

### 2.8. Immunoblotting

Western blot analysis was performed in our previous studies [18]. In brief, the hepatic tissues were homogenized and nuclear proteins were extracted with a Nuclear-Cytosol Extraction kit (Applygen Technology Inc., Beijing, China) according to the manufacturer's instructions. The protein concentration had been determined by the BCA protein assay (Bio-Rad, Hemel Hempstead, Herts, UK). Sixty micrograms of each protein sample was subjected to Western blot analysis using the following primary antibodies incubated overnight at 4°C: anti-gp91^phox^ at 1 : 8000 dilution (Cell Signaling Technology Inc.), anti-p47^phox^ at 1 : 1000 dilution (Cell Signaling Technology Inc.), anti-Na/K-ATPase at 1 : 1000 dilution (Cell Signaling Technology Inc.), anti-cleaved caspase-3 at 1 : 1000 dilution (Cell Signaling Technology Inc.), anti-procaspase-3 at 1 : 1000 dilution (Cell Signaling Technology Inc.), anti-nuclear factor kappa B (NF-*κ*B) p65 at 1 : 1000 dilution (Cell Signaling Technology Inc.), anti-Toll-like receptor 4 (TLR4) at 1 : 1000 dilution (Santa Cruz Biotechnology Inc.), and anti-*β*-actin at 1 : 1500 dilution (Merck Millipore, Germany). The secondary antibodies were goat anti-mouse or anti-rabbit IgG antibodies at 1: 2000 dilution (Thermo Fisher Scientific, Fremont, CA, USA). The enhanced chemiluminescence system was used to detect the protein-antibody complex (KGP1125, Nanjing KeyGEN Biotech. Co. Ltd.). The AlphaView software (Cell Biosciences, Santa Clara, CA) was used to measure the optical density of the interesting protein band signals which were correlated to the protein levels and normalized to those of *β*-actin.

### 2.9. Analysis of Data

Data were expressed as means ± SD. Statistical significance among groups was determined by one-way ANOVA followed by Newman-Keuls post hoc analysis using the GraphPad Prism 6 software (San Diego, CA, USA). Statistical significance was accepted at *P* < 0.05.

## 3. Results

### 3.1. Propofol Postconditioning Reduced Liver Injury after OALT

**Figure 1:**
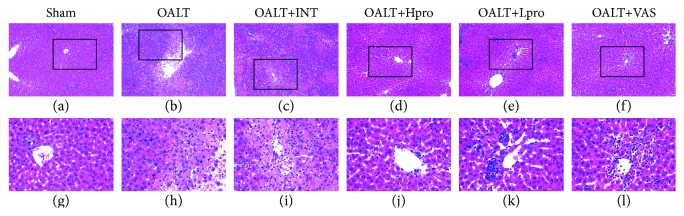
Representative photomicrographs of the livers after 8 hours of OALT. The liver tissue sections were stained with hematoxylin and eosin (H&E staining, 100x and 400x). *n* = 8 per group. ^#^*P* < 0.01 vs. the sham group; ^∗^*P* < 0.05 and ^∗∗^*P* < 0.01 vs. the OALT group. HPro = high dose of propofol; LPro = low dose of propofol; INT = intralipid; VAS = VAS2870; OALT = orthotopic autologous liver transplantation.

**Figure 2:**
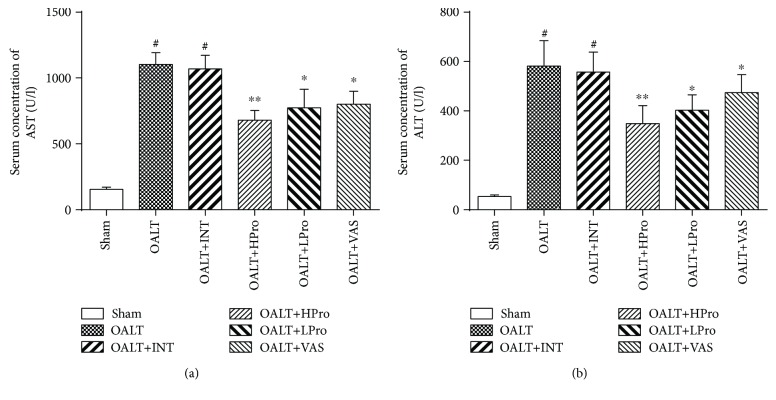
Serum alanine aminotransferase (ALT) (a) and aspartate aminotransferase (AST) (b) levels of experimental rats 8 hours after OALT. The results are expressed as the mean *±* SD. *n* = 8 per group. ^#^*P* < 0.01 vs. the sham group; ^∗^*P* < 0.05 and ^∗∗^*P* < 0.01 vs. the OALT group. HPro = high dose of propofol; LPro = low dose of propofol; INT = intralipid; VAS = VAS2870; OALT = orthotopic autologous liver transplantation.

As shown in Figure 1, compared with the sham group, there was a massive cellular necrosis (Table 1) in the centrilobular regions of the livers at 8 hours after OALT, accompanied with severe cell ballooning and infiltration of inflammatory cell, which was assessed and scaled according to the modified Suzuki criteria (*P* < 0.01 vs. the sham group). Propofol postconditioning, especially administrated at high dose (40 mg/kg/h), significantly reduced the extent of necrosis, cell ballooning, and inflammatory cell infiltration (*P* < 0.01 vs. the OALT group or intralipid group). Similarly, the Nox2 inhibitor VAS2870 exerted the same protective effects in the livers against I/R injury following OALT, evidenced by ameliorated cell necrosis, cell ballooning, and inflammatory cell infiltration (*P* < 0.05 vs. the OALT group). Consisted with the pathological results, as shown in Figures 2(a) and 2(b), high dose of propofol dramatically attenuated AST and ALT levels compared with the OALT group or intralipid group. These results indicated that propofol postconditioning and Nox2 inhibition could both provide liver protection in the early stage of OALT.

### 3.2. Nox2 Inhibition Was Involved in the Protective Effects Conferred by Propofol Postconditioning

**Figure 3:**
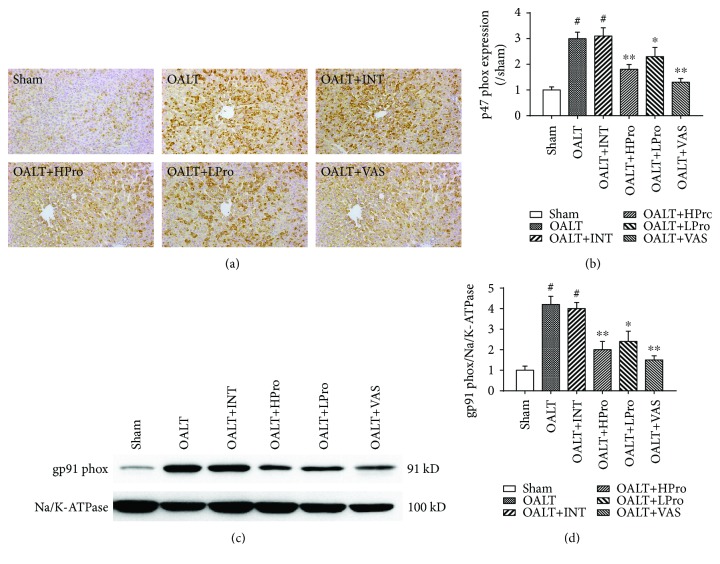
Hepatic NADPH oxidase expression changed due to OALT. Protein p47^phox^ expression was detected by immunohistochemistry method (a). The amount of protein p47^phox^ was calculated by gray scanning and was analyzed (b). Protein gp91^phox^ expression was detected by Western blot method (c). The amount of protein gp91^phox^ was calculated by gray scanning and was analyzed (d). The results are expressed as the mean ± SD. *n* = 8 per group. ^#^*P* < 0.01 vs. the sham group; ^∗^*P* < 0.05 and ^∗∗^*P* < 0.01 vs. the OALT group. HPro = high dose of propofol; LPro = low dose of propofol; INT = intralipid; VAS = VAS2870; OALT = orthotopic autologous liver transplantation.

In order to test whether the antioxidative effect of propofol postconditioning was linked to Nox2, the Nox2 subunits p47^phox^ on cell membrane and gp91^phox^ in cytoplasm were detected. As shown in Figures 3(a)–3(d), OALT leads to upregulation of p47^phox^ and gp91^phox^ protein expressions, while propofol postconditioning significantly decreased these two Nox2 subunit protein expressions in the liver following OALT. Moreover, Nox2 specific inhibitor VAS2870 was used as a positive control showing dramatically inhibition of p47^phox^ and gp91^phox^ protein expressions after VAS2870 treatment. Taken together, these results revealed that propofol postconditioning may reduce hepatic oxidative stress via inhibiting NADPH oxidase Nox2 activity and the subsequent ROS generation.

### 3.3. Propofol Postconditioning Attenuated Liver Inflammatory Response following OALT

**Figure 4:**
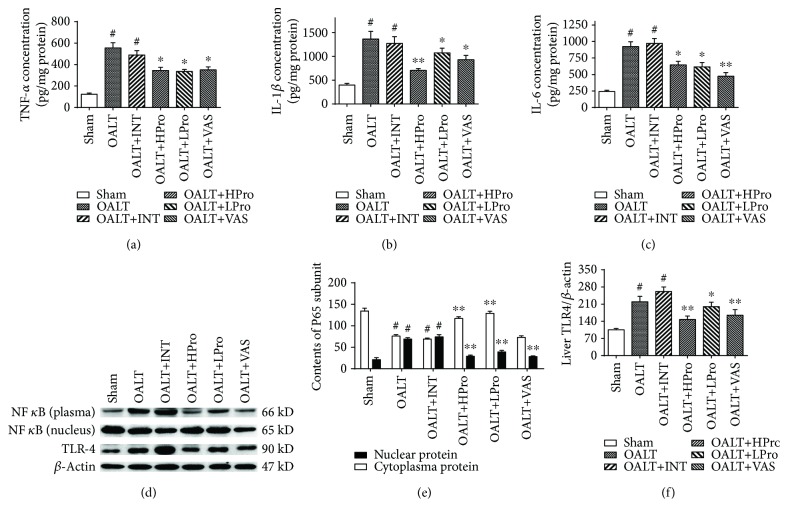
Hepatic inflammatory response after OALT. Proinflammatory cytokines TNF-*α* (a), IL-1*β* (b), and IL-6 (c) levels were measured by ELISA assay. Cytoplasm and nuclear NF-*κ*B p65 and TLR4 proteins were detected by Western blot (d). The amount of target proteins NF-*κ*B p65 (e) and TLR4 (f) was calculated by gray scanning and was analyzed. *n* = 8 per group. ^#^*P* < 0.01 vs. the sham group; ^∗^*P* < 0.05 and ^∗∗^*P* < 0.01 vs. the OALT group. HPro = high dose of propofol; LPro = low dose of propofol; INT = intralipid; VAS = VAS2870; OALT = orthotopic autologous liver transplantation.

According to the pathological results, we found inflammatory infiltration during liver injury in the early stage of OALT; we then tested the inflammatory cytokines and inflammation-related TLR4/NF-*κ*B pathway. As shown in Figures 4(a)–4(c), hepatic proinflammatory cytokines TNF-*α*, IL-1*β*, and IL-6 were all increased in the OALT group. Propofol postconditioning of both doses but not intralipid treatment significantly reduced the releases of proinflammatory cytokines compared to the OALT group. Treatment with Nox2 inhibitor VAS2870 presented similar anti-inflammation effects with decrease of levels of cytokines TNF-*α*, IL-1*β*, and IL-6. Both propofol postconditioning and Nox2 inhibition could inhibit the TLR4/NF-*κ*B inflammatory pathway, evidenced by reduced nuclear protein expressions of NF-*κ*B p65 protein and downregulated total TLR4 expression (Figures 4(d)–4(f)) (*P* < 0.05 vs. OALT).

### 3.4. Propofol Postconditioning Mitigated Neutrophil Infiltration and Hepatic Oxidative Stress

**Figure 5:**
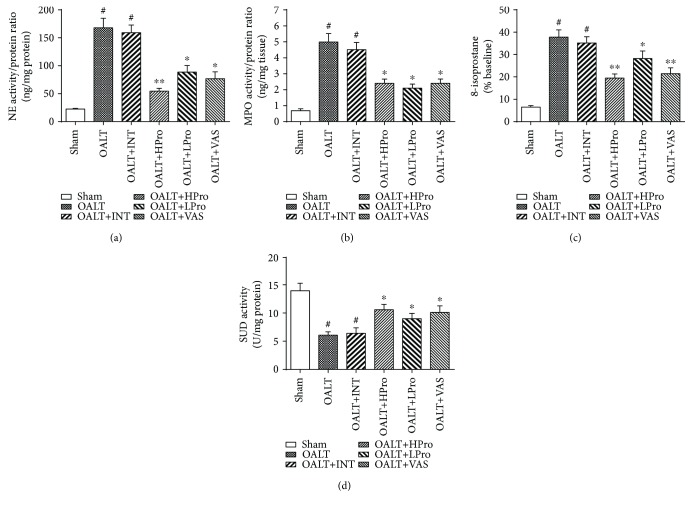
Neutrophil infiltration and oxidative stress in the liver. Neutrophil elastase (NE) (a) and myeloperoxidase (MPO) (b) activities reflected neutrophil infiltration. 8-Isoprostane (c) and superoxide dismutase (SOD) (d) were detected to reflect hepatic oxidative stress level. *n* = 8 per group. ^#^*P* < 0.01 vs. the sham group; ^∗^*P* < 0.05 and ^∗∗^*P* < 0.01 vs. the OALT group. HPro = high dose of propofol; LPro = low dose of propofol; INT = intralipid; VAS = VAS2870; OALT = orthotopic autologous liver transplantation.

ROS scavenging is one of the characteristics of propofol. In order to detect the antioxidative effects of propofol postconditioning on liver I/R injury after OALT, neutrophil infiltration and hepatic oxidative stress were measured. As shown in Figures 5(a) and 5(b), hepatic neutrophil elastase (NE) activity and MPO activity, both of which were associated with neutrophil infiltration, were significantly elevated in rats subjected to OALT. Both propofol postconditioning and VAS2870 treatment inhibited neutrophil infiltration caused by OALT and reduced lipid peroxidation product 8-isoprostane generation and increased SOD activity (Figures 5(c) and 5(d)) in the livers (*P* < 0.05 vs. OALT). These results indicated that propofol postconditioning and Nox2 inhibition protected the liver from oxidative stress via reduced neutrophil infiltration and ROS generation.

### 3.5. Propofol Postconditioning Protected Hepatocytes from Apoptosis

**Figure 6:**
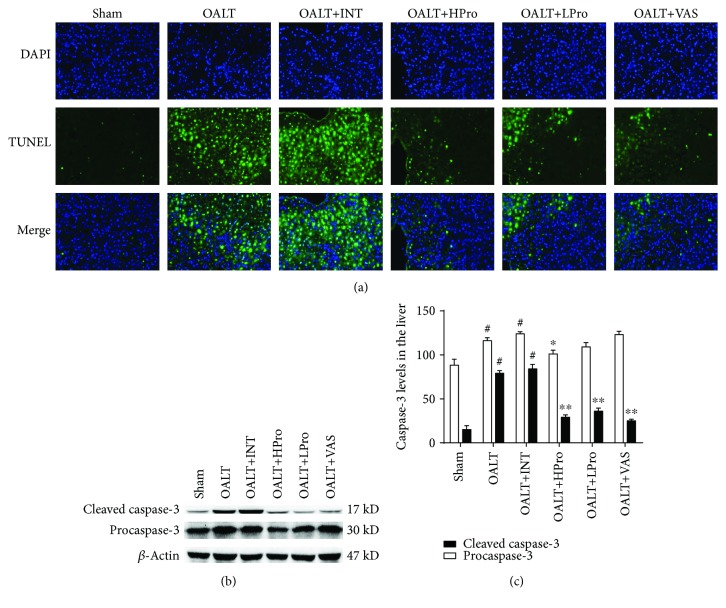
Propofol postconditioning protected hepatocyte from apoptosis. Fluorescent TUNEL staining of liver tissue (40x) (a). Cleaved caspase-3 and procaspase-3 proteins were detected by Western blot method (b). The amount of target proteins cleaved caspase-3 and procaspase-3 was calculated by gray scanning and was analyzed (c). The results are expressed as the mean ± SD. *n* = 8 per group. ^#^*P* < 0.01 vs. the sham group; ^∗^*P* < 0.05 and ^∗∗^*P* < 0.01 vs. the OALT group. HPro = high dose of propofol; LPro = low dose of propofol; INT = intralipid; VAS = VAS2870; OALT = orthotopic autologous liver transplantation.

Oxidative stress and inflammation can finally lead to hepatocyte apoptosis or necrosis and cause liver I/R injury. As shown in Figures 6(a)–6(c), we identified a significant amount of cell apoptosis occurred in the liver following OALT compared to the sham group (*P* < 0.01 vs. the sham group). High dose of propofol postconditioning significantly reduced the number of apoptotic cells, which was consistent with the decrease of cleaved caspase-3/procaspase-3 ratio. Similarly, Nox2 inhibition by VAS2870 reduced hepatocyte apoptosis compared to the OALT group. These results indicated that propofol postconditioning reduced hepatocyte ROS generation and finally protected hepatocyte from apoptosis.

## 4. Discussion

**Figure 7:**
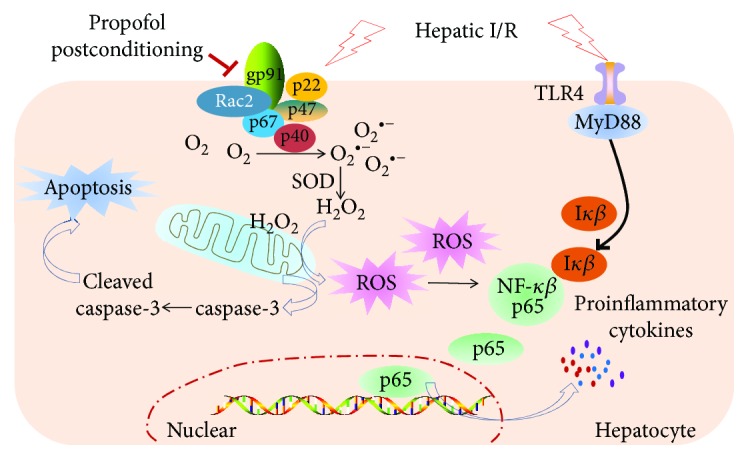
Propofol postconditioning reduces liver injury and the possible mechanisms. Under hepatic I/R condition, NADPH oxidase and TLR4/NF-*κ*B pathway are activated. Endogenously, ROS were generated due to NADPH oxidase activation, resulting in the caspase-3-related apoptosis pathway as well as NF-*κ*B pathway activation. Amount of proinflammatory cytokines was produced after NF-*κ*B p65 pathway activation. Propofol postconditioning inhibited Nox2 (gp91^phox^ and p47^phox^) which could lead to downregulation of ROS generation and finally reduced hepatic I/R injury.

In the current study, we demonstrated that propofol postconditioning reduced hepatocellular apoptosis after liver transplantation and its antioxidative property was related to inhibition of NADPH oxidase. We established an OALT model to mimic clinical liver transplantation and then detected the hepatic pathology, oxidative mediators, and inflammation response including TLR4/NF-*κ*B signaling pathway. To further clarify the protective effects of propofol, VAS2870, a specific inhibitor of NADPH oxidase Nox2, was used as a positive control. Our results suggested that propofol postconditioning exerted protective effects against liver injury following OALT. And inhibition of Nox2 maybe a possible mechanism for liver protection conferred by propofol postconditioning (Figure 7).

Liver I/R injury has been identified as one of the most important factors to the etiology after liver transplantation, which contributes to early graft dysfunction and failure [19]. I/R processed is triggered when a donor liver is transiently deprived of oxygen and reoxygenation, leading to uncontrolled inflammatory response and reactive oxygen species release in the early stages of reperfusion [20]. In the present study, we found dramatically increased hepatic inflammatory cytokines including TNF-*α*, IL-1*β*, and IL-6 as well as activated TLR4/NF-*κ*B signaling pathway after OALT. These cytokines were proved to be vital to the initiation and propagation of liver I/R injury, whose main role was to recruit circulating neutrophils to the injured liver tissue during reperfusion [21]. In our study, hepatic MPO and neutrophil elastase were increased, which indicated that neutrophil extensively infiltrates the liver in the early stage of OALT. Along with neutrophil infiltration was excessive ROS generation and subsequent oxygen-derived product formation [22].

Strong evidences have illustrated the importance of ROS in pathogenesis of liver I/R injury [23, 24]. One of the currently promising intervention strategies is ischemic preconditioning (IPC), which is an intrinsic process whereby repeated short episode of ischemia to protect the liver against subsequent ischemia [25]. However, IPC may lead to potential vascular injury and thermogenesis [26]. We previously used propofol pretreatment during liver transplantation and found that propofol protected the lung from oxidative stress via enhancing antioxidant enzyme HO-1 expression. However, it takes several days to pretreat with propofol but most liver transplantations are emergency operations [6, 7]. Thus, we preferred propofol postconditioning and demonstrated its protective function in reducing early liver damage after transplantation. In the model of rats' middle cerebral artery occlusion, it has proved that propofol postconditioning (20 mg/kg/h for 2 hours at the onset of reperfusion) led to long-term recovery of brain functions and upregulating the activity of the PKM*ζ*/KCC2 pathway [27]. Li et al. found that propofol postconditioning enhanced cell viability and alleviated apoptosis to protect cardiomyocytes against hypoxia/reoxygenation injury through ERK signaling pathway [28]. Of interest, Li et al. found that alternative use of isoflurane and propofol conferred superior cardioprotection against postischemic myocardial injury and dysfunction, and this function was probably mediated through attenuating cardiac oxidative damage [29], which indicated that anesthesia may play an important role in organ protection during I/R injury.

NADPH oxidase activation and subsequent ROS formation are important upstream events which can activate hepatocytes and amplify the production of multiple proinflammatory cytokines, such as TNF-*α* or interlukin-1*β* [30]. Hepatic NADPH oxidase activation and the ROS production have been implicated as critical regulators of liver I/R injury [23]. Although propofol has been shown to reduce oxidative stress as an ROS scavenger, the current study shows that propofol can also suppress Nox2 to reduce the consequent production of ROS. Notably, propofol downregulated the hepatic expression of the NADPH oxidase membrane components p47^phox^ and glycosylated subunit gp91^phox^ after OALT. We speculated that this may be an important mechanism of propofol actions. Luo et al. showed that siRNA silencing of p22^phox^ significantly attenuated the protective effects of propofol [31]. Recent studies have also identified other receptors as potential molecular targets of propofol including nicotinic and M1 muscarinic receptors [32, 33]. Whether these receptors act as upstream regulators, NADPH oxidase remains to be determined.

Of note, in the current study, the OALT model is superior in mimicking the pathophysiological variation during liver ischemia/reperfusion in liver transplantation and ischemia/reperfusion-mediated liver injury without interference of immunoactivities between grafts and hosts. However, as the cold ischemia time in this model is about 20 minutes, so it is not able to represent the long (6 to 8 hours) cold preservation time that occurs in the liver graft before being transplanted. Propofol postconditioning was performed at the onset of reperfusion with continuous infusion for 30 min and proved to be protective against liver I/R injury. However, whether the dose and the duration we chose was the best intervention required further investigation. Moreover, although we have confirmed that the protective effect of propofol postconditioning was related to Nox2 activity inhibition, whether it acts on the other NADPH oxidase subunit such as Nox4 also remains unknown. It is also still unclear that how propofol acts on Nox2, directly or indirectly. Those questions remain unanswered. More studies will be involved to clarify these mechanisms and make this intervention more safe and reliable.

In summary, liver transplantation leads to severe inflammation and oxidative stress accompanied with NADPH oxidase Nox2 activation. Propofol postconditioning exerted prominently protective function against the I/R injury after liver transplantation, which presented as lower levels of inflammatory mediators and oxidative products accompanied with less neutrophil filtration and weaker induction of Nox2. Collectively, propofol postconditioning had been proved to reduce liver inflammation and oxidative stress probably via inhibiting NADPH oxidase Nox2.

## Figures and Tables

**Table 1:** Histological score of liver injury by OALT.

Groups	Centrilobular cell death					Ballooning					Inflammation				
	Grade				Average	Grade				Average	Grade				Average
	+3	+2	+1	0		+3	+2	+1	0		+3	+2	+1	0	
Sham	0	0	0	8 (100)	0	0	0	0	8 (100)	0	0	0	0	8 (100)	0
OALT	2 (25)	3 (38)	3 (38)	0 (0)	1.9^#^	3 (38)	3 (38)	2 (25)	0 (0)	2.1^#^	5 (63)	2 (25)	1 (13)	0 (0)	2.5^#^
OALT + INT	2 (25)	4 (50)	2 (25)	0 (0)	2.0^#^	2 (25)	4 (50)	2 (25)	0 (0)	2.0^#^	4 (50)	3 (38)	1 (13)	0 (0)	2.4^#^
OALT + HPro	0 (0)	1 (13)	6 (75)	1 (13)	1.0^∗∗^	0 (0)	1 (13)	7 (88)	0 (0)	1.1^∗∗^	0 (0)	2 (25)	5 (63)	1 (13)	1.1^∗∗^
OALT + LPro	0 (0)	1 (13)	7 (88)	0 (0)	1.1^∗∗^	0 (0)	2 (25)	6 (75)	0 (0)	1.3^∗^	0 (0)	4 (50)	4 (50)	0 (0)	1.5^∗^
OALT + VAS	0 (0)	2 (25)	6 (75)	0 (0)	1.3^∗^	0 (0)	2 (25)	5 (63)	1 (13)	1.1^∗∗^	0 (0)	3 (38)	4 (50)	1 (13)	1.3^∗^

Numbers of rats are shown, with percentages enclosed within parenthesis. Grade indication: no change (0), mild (1), moderate (2), and severe (3). ^#^*P* < 0.01 vs. the sham group, ^∗^*P* < 0.05 vs. the OALT group, and ^∗∗^*P* < 0.01 vs. the OALT group.

## Data Availability

The data used to support the findings of this study are available from the corresponding author upon request.
